# Rehabilitation Treatment and Progress of Traumatic Brain Injury Dysfunction

**DOI:** 10.1155/2017/1582182

**Published:** 2017-04-11

**Authors:** Baoqi Dang, Wenli Chen, Weichun He, Gang Chen

**Affiliations:** ^1^Department of Neurosurgery & Brain and Nerve Research Laboratory, The First Affiliated Hospital of Soochow University, 188 Shizi Street, Suzhou 215006, China; ^2^Department of Rehabilitation Medicine, Zhangjiagang Hospital of Traditional Chinese Medicine, Suzhou 215600, China

## Abstract

Traumatic brain injury (TBI) is a major cause of chronic disability. Worldwide, it is the leading cause of disability in the under 40s. Behavioral problems, mood, cognition, particularly memory, attention, and executive function are commonly impaired by TBI. Spending to assist, TBI survivors with disabilities are estimated to be costly per year. Such impaired functional outcomes following TBI can be improved via various rehabilitative approaches. The objective of the present paper is to review the current rehabilitation treatment of traumatic brain injury in adults.

## 1. Introduction

Traumatic brain injury (TBI) refers to blunt, penetrating, or acceleration/deceleration force-derived craniocerebral injury, which causes symptoms such as decline in level of awareness or consciousness, memory loss or forgetfulness, other neurological or neuropsychological abnormalities, and even death. TBI is a critical public health and socioeconomic problem throughout the world. The incidence of TBI has been increasing annually. According to the World Health Organization, TBI will be a major health problem and the main reason for disability in 2020 [1]. Primary and secondary TBIs cause temporary and/or permanent dysfunction in the brain, which limits a patient's activities, affects participation in society, and lowers quality of life. This can lead to depression and other chronic diseases in TBI patients [2, 3]. This article reviews current rehabilitation treatment for TBI.

## 2. Hyperbaric Oxygen Therapy (HBOT) Relieves TBI

Hyperbaric oxygen therapy (HBOT) is defined as the inhalation of 100% oxygen under the pressure greater than 1 atmosphere absolute (ATA) (1 ATA = 101.3 kPa). HBOT is a current interest in the field of neurological diseases and has been proved to inhibit apoptosis, suppress inflammation, protect the integrity of blood-brain barrier, and promote angiogenesis and neurogenesis [4, 5].

The major pathogenic mechanisms of TBI include ischemia and hypoxia in brain tissues, resulting in parenchymal softening with necrosis. To date, HBOT is one of the most important clinical therapies for TBI. A study by Lin et al. [6] showed that 2.0 atmospheres absolute (ATA) oxygen in HBOT for 5 consecutive days (once per day, 1 hour per session) resulted in overexpression of the 70 kDa heat shock protein (HSP-70) and attenuated cerebral edema, oxidative damage in the hippocampus, and cognitive impairment in a rat model in a simulated high-altitude environment (9.7% oxygen concentration, 6000 meter altitude, and 0.47 ATA) for 3 consecutive days. Harch et al. [7] treated 16 TBI, post-TBI syndrome, and posttraumatic stress disorder (PTSD) patients with 40 sessions of 1.5 ATA/60 minutes of HBOT for 30 days, which greatly improved symptoms, results of neurological examinations, comprehensive IQ tests, and cognition functions. A study by Geng et al. [8] showed that HBOT may suppress activation of inflammasome signals, thereby alleviating TBI.

In chronic brain injury, HBOT improved cerebral blood flow (CBF) and ameliorated the neuropsychological disorders [9, 10]. HBOT has also been reported to show positive effects by improving the quality of life in patients with postconcussion syndrome or mild TBI at late chronic stage [7, 11, 12]. In severe TBI, HBOT has reduced mortality and enhanced functional outcome [13–15]. These researches suggested the successful use of intensive HBO as a treatment in TBI patients.

## 3. Noninvasive Brain Stimulation Benefits the Treatment of TBI

To date, several technologies have been developed. The most common technologies are transcranial magnetic stimulation (TMS) and transcranial direct current stimulation (tDCS). Repetitive transcranial magnetic stimulation (rTMS) is a painless, noninvasive, easily operated treatment with few adverse reactions. It has specific effects in rehabilitation of TBI patients [16]. Depending on the frequency used, rTMS alters neuronal excitability by generating excitatory (>5 Hz) or inhibitory (1 Hz) activity, which can last for several hours [17, 18]. As a noninvasive brain stimulation technique, rTMS has successfully treated schizophrenia, depression, Parkinson's disease, aphasia, unilateral neglect, and cognitive impairment [19–24]. Neville et al. [25] conducted a double-blind randomized controlled trial with 36 TBI patients who were randomly and equally divided into 2 groups. TBI patients in the treatment group received 10 sessions of high-frequency rTMS (10 Hz) over the left dorsolateral prefrontal cortex (DLPFC), and TBI patients in the sham group received pseudostimulation. The patients underwent neuropsychological assessment 1 week before and then 1 week and 3 months after rTMS to evaluate direct and delayed effects. The results showed that rTMS could improve depression and cognitive function after TBI.

Dhaliwal et al. [26] and Li et al. [27] recently reported that cerebral stimulation had potential effects on TBI treatment. Although these 2 groups studied non-athletic-related injuries, they indicated the safety and potential benefits of cerebral stimulation for TBI patients. Several studies have shown that rTMS and tDCS reduce TBI-associated depression, tinnitus, neglect, memory deficits, and attention disorders [26, 27]. Middleton et al. [28] also reported that 2 TBI patients who received dual-hemisphere tDCS (1 patient had stroke and TBI) for 6 months showed significant improvement based on the upper extremity Fugl-Meyer scale. These studies demonstrate that post-TBI cerebral stimulation is safe and has potential benefits.

## 4. Virtual Reality Evaluates the Function and Improves the Prognosis of TBI

Computer-aided training combined with audial and visual stimulations to simulate audial, visual, and game-associated intuitive trainings that engage different components of impairment, such as memory, attention, and visual perception, greatly improves patient interest and enthusiasm in participation. Development of a computer-aided training system, especially virtual reality (VR) technology, promotes integration between computer technology and cognitive science that has incomparable advantages for assessment and training of cognitive impairment compared with cognitive training by rehabilitation therapists [29–31]. During the training process, personal, customized procedures are used to reduce the duration of the direct contact between the therapist and the patient. Studies have shown that computer-aided strategies improve patient attention, memory, and execution capabilities [32, 33]. VR training also improves patient mood through audial and visual feedback that lets patients experience emotional success and minimizes patient anxiety during treatment [34]. It also promotes persistence since patients practice until they succeed. In addition, a VR training system provides a comprehensive evaluation of patient motor function, cognitive function, daily life skills, and social skills. It directly analyzes the data and presents a written report for comparison of pre- and posttreatment conditions, which help in determining treatment goals, selecting treatment options, and evaluating training effects to achieve a perfect combination of interactive training and exercise, cognitive training, and rehabilitation assessment [35].

## 5. Limb or Organ Function Reconstruction following TBI

Functional electrical stimulation (FES) is a low-frequency pulse current that is used to stimulate limb or organ dysfunction. Its effects replace or correct lost function in limbs and organs. By adjusting the advanced nerve center, FES promotes functional reconstruction in patients [36]. Task-oriented functional electrical stimulation (TFES) is a combination of bilateral exercise, repetitive training, task-oriented therapy, and FES, and preliminary results have indicated positive outcomes [37–40].

Treatment outcomes of TFES are better than those of FES and conventional therapy. A possible explanation may combine the effects of FES and task-oriented therapies as well as the synergistic effect of this combination therapy. Iftime-Nielsen et al. [41] confirmed a synergistic effect between proactive attitude of patients and FES therapy. Makowski et al. [42] showed that a combination of FES therapy and conscious activity affected action stimulated by FES. Under the effect of FES, a weak and voluntary effort can produce greater reach and movement.

Calabrò et al. [43] conducted 2 different types of intensive rehabilitation training for a 34-year-old male with dysphagia after TBI, which included conventional rehabilitation training and a combination of conventional rehabilitation training and VitalStim electrical stimulation therapy for 6 weeks to access his specific swallowing function and electrophysiological parameters before and after treatment. The results showed that only VitalStim point simulation significantly improved the swallowing function of the patient. This patient could eventually and safely eat solid food after the treatment.

## 6. TBI Benefits from Behavioral, Emotional, and Family Therapies

TBI affects a patient's emotions, behavioral stability, and self-confidence. Primary caregivers of TBI patients experience considerable emotional stress and sense of burden. Albert et al. [44] showed that low-cost interventions relieve the burden of caregivers and improve their satisfaction. A study by Sinnakaruppan et al. [45] showed that family education programs for caregivers and TBI family members help relieve stress and strengthen coping abilities.

Common behavioral changes after TBI often include anger, depression, anxiety, and verbal or physical aggression. Emotional stability of TBI patients is necessary; otherwise, these patients cannot participate in and benefit from the rehabilitation processes. Psychotherapy (individual and group) emphasizes emotional and behavioral therapies. Studies show that training in good coping skills and anger management can reduce patient aggression. In addition, Baker et al. [46] showed that music therapy demonstrated an improvement in patient emotion and anger problems.

## 7. Basic Research on TBI Rehabilitation in Recent Years

In recent years, the basic research on traumatic brain injury rehabilitation increase gradually [47–51]. A recent review suggests that rat models and closed head impacts have dominated the field of behavioral testing in animal models of juvenile TBI. Both motor and cognitive functions seem to be affected [47]. A latest study revealed that long-term spatial learning-memory deficits are dependent on the severity of destruction in the white matter and hippocampus. Therapeutic strategies targeting both the white matter and hippocampus may be needed to improve the neurological functions in TBI victims [50].

Studies have shown that the major cause of death after TBI is neuronal death and rupture of blood vessels. Nerve regeneration and angiogenesis play key roles in functional recovery [52–54]. Circulating endothelial progenitor cells (EPCs) are involved in angiogenesis [55, 56] and have been confirmed to reduce infarct volume, increase capillary density, and improve myocardial blood perfusion and limb ischemia in animal models [57, 58]. Erythropoietin (EPO) promotes proliferation and differentiation of red blood cells and has been used in clinical treatment of anemia, prevention of spinal cord injury [59], retinal ischemia [60],skeletal muscle ischemia [58],pulmonary hypertension [61], and myocardial ischemia-reperfusion injury [62, 63]. It is also used for prevention of TBI by enhancing antiapoptotic [59, 64], anti-inflammatory [60], and neuroprotective effects [65, 66]. Through mobilization of endothelial progenitor cells, EPO promotes angiogenesis and reduces nerve cell death, which improves functional outcomes after stroke [67–69]. Data of Wang et al. [49] showed that recombinant human EPO mobilized endothelial progenitor cells and angiogenesis to improve the functional prognosis of TBI in rats.

In summary, rehabilitation is essential after TBI treatment. Studies on the timing of corresponding rehabilitation in stroke research are common. However, the optimal window for TBI rehabilitation is rarely reported. Andelic et al. [70] divided 61 patients with severe TBI into 2 groups: the experimental group with early intervention of rehabilitation training and the control group with delayed rehabilitation training. The Glasgow Outcome Scale Extended (GOSE) and the Disability Rating Scale (DRS) were used to rate the 2 groups 12 months after training. The results demonstrated that the experimental group had significantly higher GOSE and DRS scores compared with the control group, which indicated that early intervention of rehabilitation training achieved better treatment outcomes. However, due to the complexity of TBI, an inadequate sample size, and lack of an appropriate control group, clinical rehabilitation studies have encountered significant challenges. For noninvasive brain stimulation, stimulation frequency, precise positioning, and course of treatment are closely associated with treatment efficacy. A large-scale randomized controlled trial is necessary in further studies, and additional research in this direction will extend the rehabilitation prospects of TBI.
